# Synthesis and analyses of injectable fluoridated-bioactive glass hydrogel for dental root canal sealing

**DOI:** 10.1371/journal.pone.0294446

**Published:** 2023-11-27

**Authors:** Nadia Irshad, Noureen Jahanzeb, Alanood Alqasim, Raneem Bousaleh, Maha Almehrij, Sarah Ghafoor, Muhammad Nawaz, Sultan Akhtar, Asma Tufail Shah, Abdul Samad Khan

**Affiliations:** 1 Department of Dental Materials, University of Health Sciences, Lahore, Pakistan; 2 Department of Dental Materials, Sharif Medical and Dental College, Lahore, Pakistan; 3 College of Dentistry, Imam Abdulrahman Bin Faisal University, Dammam, Saudi Arabia; 4 Dental and Oral Health Department, Prince Sultan Military College of Health Sciences, Dhahran, Saudi Arabia; 5 Department of Oral Biology, University of Health Sciences, Lahore, Pakistan; 6 Department of Nano-Medicine Research, Institute for Research and Medical Consultations (IRMC), Imam Abdulrahman Bin Faisal University, Dammam, Saudi Arabia; 7 Department of Biophysics, Institute for Research and Medical Consultations (IRMC), Imam Abdulrahman Bin Faisal University, Dammam, Saudi Arabia; 8 Interdisciplinary Research Centre in Biomedical Materials (IRCBM), COMSATS University Islamabad, Lahore Campus, Pakistan; 9 Department of Restorative Dental Sciences, College of Dentistry, Imam Abdulrahman Bin Faisal University, Dammam, Saudi Arabia; University of Minnesota, UNITED STATES

## Abstract

This study aimed to synthesize fluoride-doped bioactive glass (F-BG) based thermo-sensitive injectable hydrogel for endodontic applications. The structural and phase analyses were done with Fourier Transform Infrared spectroscopy and X-ray Diffraction, respectively. The setting time of prepared injectable was investigated at 21°C (in the presence and absence of an ultrasonic scalar) and at 37°C. Flowability was tested according to ISO-6876:2012 specifications, whereas injectability was checked by extrusion method using 21-, 22-, and 23-gauge needles. The *in vitro* bio-adhesion and push-out bond strength were studied on days 7 and 90 and compared with the commercially available TotalFill®. The ion release profile was analyzed for up to 30 days with Inductively Coupled Plasma Optical Emission Spectroscopy. The fluoride release analysis was conducted periodically for up to 21 days in deionized water and artificial saliva using an ion-selective electrode. The final setting time at 21°C, 21°C+ultrasonic scalar, and 37°C were 38.66±3.21, 29.12±1.23, and 32±3.46 min, respectively. The flowability was 25±3.94 mm, and the injectability coefficient was ≥70.3 for 22, 21, and 57% in a 23-gauge needle. Fluoride release in deionized water was found to be significantly higher than in artificial saliva and increased with time. A significant difference in bond strength was found between days 7 and 90, where the strength was increased, and a new apatite layer was formed on the tooth surface. A rapid release of calcium, phosphate, and silicon ions was seen initially, whereby the continuous release of these ions was observed for up to 30 days. The prepared F-BG injectable hydrogel has shown promising results and has the potential to be used as an endodontic sealer.

## Introduction

As per the oral health status report published by WHO in 2022, oral disease affects almost 3.5 billion people across the world, whereby 2 billion individuals suffer from caries in permanent teeth, and nearly 514 million children have caries in deciduous teeth [1]. The progression of dental caries leads to necrosis of the pulp, subsequently to periapical lesion formation [2]. The best treatment option in such cases is the root canal or endodontic treatment. The endodontics market size exceeded U.S $ 1.1 billion in 2022 and is expected to increase at an annual growth rate of 4% between 2023 to 2032 [3] A study reported that the global prevalence of the root canal treated teeth was 55.7% where the European population has shown the highest prevalence [4]. A reported success rate of primary root canal treatment is 68–85%, where endodontic treatment aims to seal the root canals as hermetically as possible to prevent microleakage. Endodontic treatment failures are directly related to apical percolation due to insufficient sealing [5].

Bioceramics such as bioactive glass, hydroxyapatite, calcium phosphates etc., have been used for restorative and dental use [6,7] and are classified as bioactive, and biodegradable materials. Bioactive materials were brought to endodontics in the 1990s, first as retrograde filling materials and then as root restoring cements, root canal sealers, and coatings for gutta‐percha cones [8,9].

Bioactive sealers have many advantages over conventional sealers, such as releasing calcium ions and producing calcium hydroxide and apatite on the dentin wall, consequently increasing the sealing ability [9]. The first endodontic product (ProRoot MTA, Dentsply, USA) based on bioactive material was marketed in 1997. Since then many endodontic sealers based on calcium silicate and calcium phosphate have been commercialized [10].

Bioactive materials such as bioactive glasses (BG) have been used for dental applications such as implant coating, bone grafts, restorative materials, and tissue engineering scaffolds [11–14,18,21]. Since the invention of BG in 1969, improvisations have been done in the chemical composition of BG by doping with various ions, such as zinc, magnesium, copper, silver, strontium, boron, fluoride [14]. Fluoride-substituted bioactive glass (F-BG) forms fluorapatite, which is more acid-resistant than hydroxyapatite [15]. F-BGs cause reduced pH rise in the surrounding area as compared to non-fluoridated glasses, which is beneficial for tissue regeneration [16].

Maintaining close contact with the host tissue is the main challenge in achieving good osteoconduction. This shortcoming of intraoperative moldability can be overcome by synthesizing injectable BGs hydrogels that can conform to any shape and size *in vitro* and *in vivo*. Injectables are becoming particularly attractive because of their minimally invasive and easy delivery to the host site [17]. Most commonly used endodontic sealer systems are either base/catalyst or powder/liquid [18]. Recently, injectable root canal sealers are marketed with improved handling properties [19–21]. When developing a novel endodontic sealer utilizing bioactive materials, careful consideration must be given to their physicochemical and bioactive properties [22], particularly their flowability and setting time [23]. Currently, for injectable bioactive ceramic pastes, there is a lack of standardization regarding the optimal setting time or mechanical strength. Therefore, an insight into the setting time, injectability, particle size, and the amount of fluoride ions release into the external environment should be considered to design and fabricate an ideal injectable material [24]. Injectable endodontic sealers possess the unique ability to flow effortlessly along the root canal, effectively reaching areas that are inaccessible to conventional paste or powder-based sealers [25]. Previously, polyurethane, sodium alginate, and hyaluronic acid-based injectables have been used in endodontics [26]. However, in the present study, Pluronic F127 has been used as polymer base. Pluronic F127 is a thermo-responsive polymer and has been used as a drug carrier [27]. It is in liquid state at room temperature and gelled at 37°C. Hydroxypropyl methylcellulose (HPMC) as a copolymer has been used as an oral medicament [28]. In order to get the benefits of these polymers and F-BG, this study was aimed to develop a novel injectable fluoride-releasing bioactive glass based on nano-sized F-BG and Pluronic F127. The properties of the material relevant to its possible clinical applications, such as injectability, setting time, and fluoride release, were studied. The current work was also aimed to rationalize and support the optimal diameter and length of needles needed for injectable bioactive glass composites. Furthermore, the *in vitro* bond strength and bio-adhesion of the experimental injectable bioactive glass-based root canal sealer was evaluated with the conventionally used bioceramics sealer.

## Materials and methods

### Synthesis of injectable

The F-BG with compositions (mol.%) 46SiO_2_: 28.5-xCaO: 23Na_2_O: 2.5P_2_O_5_: 5NaF was prepared by base-catalyzed sol-gel method (co-precipitation method) as previously described by our group [29]. The surface area and average pore size of F-BG particles was 64.3 m^2^.g^-1^ and 8 nm, respectively. Thermo-sensitive injectable bone substitute cement was prepared by using non-ionic triblock copolymer pluronic F127 (E106 P70 E106) (Sigma Aldrich, St. Louis, MO, USA) and hydroxypropyl methylcellulose (HPMC) (Sigma Aldrich, St. Louis, MO, USA) as carrier, and F-BG as filler. In brief, 0.2 g of triblock copolymer pluronic F127 was dissolved in 1.2 mL phosphate buffer solution (PBS) at 4°C. After 30 min, 0.4 g F-BG was slowly added into the polymeric solution and stirred for 1 h at 250 RPM. Then 0.18 g HPMC was added and mixed until it became a homogenous suspension. This suspension was continuously stirred to avoid any precipitation. The prepared injectable material was stored in a glass vial in a refrigerator at 4°C [30].

## Characterizations

### Setting time

The initial and final setting time of the injectable F-BG was measured by Gilmore needle apparatus as per ASTM C266-99 specifications [31] at two different temperatures i.e., 21 and 37° [32]. The setting time of the hydrogel at 37°C (that mimic the body temperature) was measured by placing the sample on a flat plastic surface and incubating it in an oven (WiseVen, DAIHAN Scientific Co., Seoul, South Korea) at 37°C. The setting time was assessed at 21°C (room temperature), both with and without the application of the ultrasonic scalar (UDS-J, GUILIN Woodpecker, Yixing, China). For measuring the setting time under ultrasonic stimulation, the injectable sample was placed on a flat plastic surface measuring 10 mm in diameter and stimulated with ultrasonic scalar (10 Hz) for 20 s intervals (to prevent heating of the scalar). The indentations were done after 5 min intervals to observe the initial and final setting time of the samples (all groups) in triplicate. The initial and final setting time was recorded. The setting of samples was evaluated in three different conditions, and a description of respective groups is given in Table 1.

**Table 1:** The sample groups based on different temperature conditions.

Group	Temperature	Stimulation	Labeling
A	21°C	None	IBG-21
B	21°C	Ultrasonic scalar	IBG-21s
C	37°C	Incubator	IBG-37

### Chemical analysis

Fourier Transform Infrared Spectroscopy (FTIR) was carried out using Thermo Nicolet 6700 (Thermo Fisher Scientific, Waltham, MA, USA) in conjunction with Attenuate Total Reflectance (ATR) to investigate the structural changes in the injectable F-BG before and after setting (21°C). The resolution was set at 8 cm^-1^ with 128 scan numbers, and spectra were taken in 4000–550 cm^-1^ range. The comparative spectra were analyzed by OMNIC^TM^ software (Thermo Fisher Scientific, USA), and the difference and shifting of peaks were observed.

#### Morphological analysis

The surface morphology of set injectable F-BG samples (at 21°C with and without ultrasonic scalar) was examined using a Scanning Electron Microscope (VEGA-3 LMU, TESCAN, Brno, Czech Republic). Before the scanning procedure, the sample was gold coated in a gold sputter coater (QUORUM Technologies, Lewes, UK) for 90 s. Images were taken in different magnifications and were operated at an accelerated voltage of 20 kV. The particle size and pore size were measured using the ImageJ software (Image J Launcher; https://imagej.net/Launcher).

#### Phase analysis

X-ray diffractometer (PANanalytical XPERT-PRO, USA) operated at 40 kV and 40 mA using Cu Kα radiation was used to determine any crystalline phases present in the injectable F-BG sample after setting. The detector was scanned over a range of angle 2θ = 10–60° at a step size of 0.02°.

#### Flowability test

Flowability was tested by using the method recommended by ISO 6876:2012. A total of 50 mg of experimental sealers (n = 10) was placed onto a glass. After three minutes, another glass plate was applied centrally on top of the material to make a total mass on the plate of 120 g. Ten minutes after the application, the load was removed, and an average of the major and minor diameters of the compressed discs was measured using a digital caliper.

#### Injectability test

The injectability of the F-BG paste was evaluated using an extrusion test. The test was conducted at room temperature with a relative humidity of 55±5%. For the test, a six-inch-long plastic hollow tube having a diameter of 2.5 cm was used, and a window of about 1.5 inches was cut into the lower half of the plastic tube in order to observe the flowability of the injectable material. A 3 mL syringe was inserted into the tube with the sides of the syringe resting on the rim of the hollow tube, and a metal holder was used to hold the plastic tube with the syringe containing a freshly prepared sample of injectable F-BG. The material (2 mL) was extruded from the syringe by applying a force using universal testing machine (Testometric M500-50AT, Rochdale, UK) at 0.5 mm.min-1 crosshead speed. The extrusion was stopped once the applied force reached 100 N [25]. Three different gauge needles, i.e., 21, 22, and 23, were used to evaluate the flowability. Three tests (n = 3) for each needle gauge were performed, and their results were expressed as mean and standard deviation for all the test specimens. The injectability coefficient (I) was calculated according to the formula mentioned previously [33]. The ease of injectability was scored by implementing a scoring method, as reported by Cilurzo [34].


$$I=[(M_{0}–M)/M_{0}]\times 100$$ (Eq 1)

Where, **Mo** is the initial mass of the cement loaded into the syringe and **M** is the mass remaining in the syringe after extrusion.

#### Bio-adhesion analysis

After taking the ethical permission from the CUI Lahore Campus Ethical Committee (EC/ATS/002/19), a total of 40 non-carious single-rooted extracted human mandibular premolar teeth were collected. The teeth were collected from the Oral and Maxillo-facial department of the institutional hospital. The teeth were extracted for orthodontic treatment. The obtained teeth were disinfected in 70% ethanol solution then preserved in 0.5% thymol solution for a month. Later, all teeth were decoronated from the cemento-enamel junction (CEJ) using a high-speed carbide bur (Syndent tools Co., Jiangsu, China) under water spray. The root canal preparation was done as described previously [35]. The prepared canals were randomly distributed into two main groups (n = 20), control Total Fill (TF; FKJ Dentarire SA, Switzerland), and experimental injectable F-BG (0.3 mL), that were injected in the respective canals. TF was used as per the manufacturer’s instructions. Canals were obturated with 0.4 taper gutta-percha (Sure-Endo Co., Ltd, South Korea) using the cold lateral compaction technique (master + accessory cones), and the surface of gutta-percha was coated with each experimental and control sealers. A layer of self-cured glass ionomer cement (Fuji IX-GP, GC America) was placed on top of the master cone to create a coronal seal. The prepared canals were evaluated with Gendex radiographic machine analysis (Dentsply Ltd, Surrey, UK).

#### Push-out bond strength analysis

The obturated roots (as mentioned above) of both groups were equally divided into two groups and placed in deionized water at 37°C for 7 and 90 days. At each specific time, the roots were cut using a precision saw cutting machine (IsoMet 5000, Lake Bluff, IL, USA) into 2 mm slices (after removal of the top 1 mm slice) and then gutta-percha was pushed out using uniaxial materials testing machine (Instron 8871; Instron, Norwood, MA, USA). A flat-ended stainless-steel jig of 0.6 mm in diameter was used to push out gutta percha from the roots. The crosshead speed was set at 0.5 mm/min. After removing the gutta percha, the slices were viewed under scanning electron microscope (SEM; FEI, Eindhoven, The Netherlands) with Energy Dispersive Spectroscopy (EDS) (SEM/EDX; TESCAN VEG3, Berno, Czech Republic). For SEM analysis, a sputter coating machine (Quorum Technologies, Lewes, UK) was used for gold coating. All samples were gold sputtered for 90 s, and images were taken at an electron beam voltage range from 5 to 20 kV.

#### Ion release analyses

The specimens (n = 10) of the experimental and control sealers were allowed to be set in a moist environment for 1 day at 37°C after obturation. Each sample was hung straight into 1.5 mL deionized water (epidorff tubes were used) after setting and placed at 37°C. The coronal part of the root was not immersed in water. The media were collected at days 1, 7, 21, and 30 and refreshed with deionized water at each time interval. The extracting media was centrifuged at 3500 rpm for 30 min, and the leaching profile of calcium, phosphate, and silicon ions were analyzed with inductively coupled plasma optical emission spectroscopy (ICP-OES; PerkinElmer AvioR 145 500 ICP-OES Scott/Cross-Flow, Waltham, MA, USA). Each sample was analyzed in triplicate.

#### Fluoride release analysis

For fluoride release analysis, sample discs (n = 7) for each media were formed by injecting the material in custom-made brass molds [5 mm (diameter) × 2 mm (height)] and allowed to set completely at 37°C. Each sample was placed in 10 mL of deionized water and 10 mL of artificial saliva, separately. The artificial saliva was prepared as described previously [36]. Samples were kept in an incubator (DAIHAN Scientific Horizontal Flow, WOF-155, Seoul, South Korea) at 37°C and were taken out from the incubator after 6 h and centrifuged in a benchtop centrifuge (Centurion Scientific, Chichester, UK) at 3500 RPM for 20 min each. Clear solution at the top of the tubes was collected in separate falcon tubes. Fresh media was replaced in the sample tubes and were placed back in the incubator at 37°C. Similar procedure was done periodically at days 1, 3, 5, 7, 14, and 21. The elutes taken on these specific days were then analyzed for fluoride release using Ion Selective Electrode (ISE) meter (HI-4110 with Hanna pH/ISE meter HI-3222; Hanna Instruments, Woonsocket, RI, USA) after the addition of 1 mL of TISAB III (HANNA instruments, Woonsocket, RI, USA) in each sample [37].

#### Statistical analysis

The statistical analysis was performed using SPSS version 22 (IBM Software, Armonk, NY, USA). The Shapiro–Wilk test was used to test the normal distribution of data. An independent t-test and one-way analysis of variance (ANOVA) were used to compare the setting time, injectability, bond strength, and ion leaching between the groups, followed by Tukey’s post-hoc test. For fluoride release analysis independent t-test and repeated measurement analysis were conducted, followed by a one-way ANOVA post hoc Tukey test, where the level of significance was set at 0.05.

## Results

### Setting time

The setting time values of three replicates were used to assess the initial and final setting time measurements for each injectable sample. The initial setting time of injectable F-BG hydrogel was 21 ± 2.8 min for IBG-21, while the final was 38.66 ± 3.21 min. The initial and final setting time for IBG-21S was 14.3 ± 2.0 min and 29.12 ± 1.23 min, respectively. In comparison, the initial and final setting time of IBG-37 was 12.3 ± 3.21 and 32 ± 3.46 min, respectively. There was a statistically significant difference (p = 0.0133) among the three groups.

### Chemical analysis

**Fig 1:**
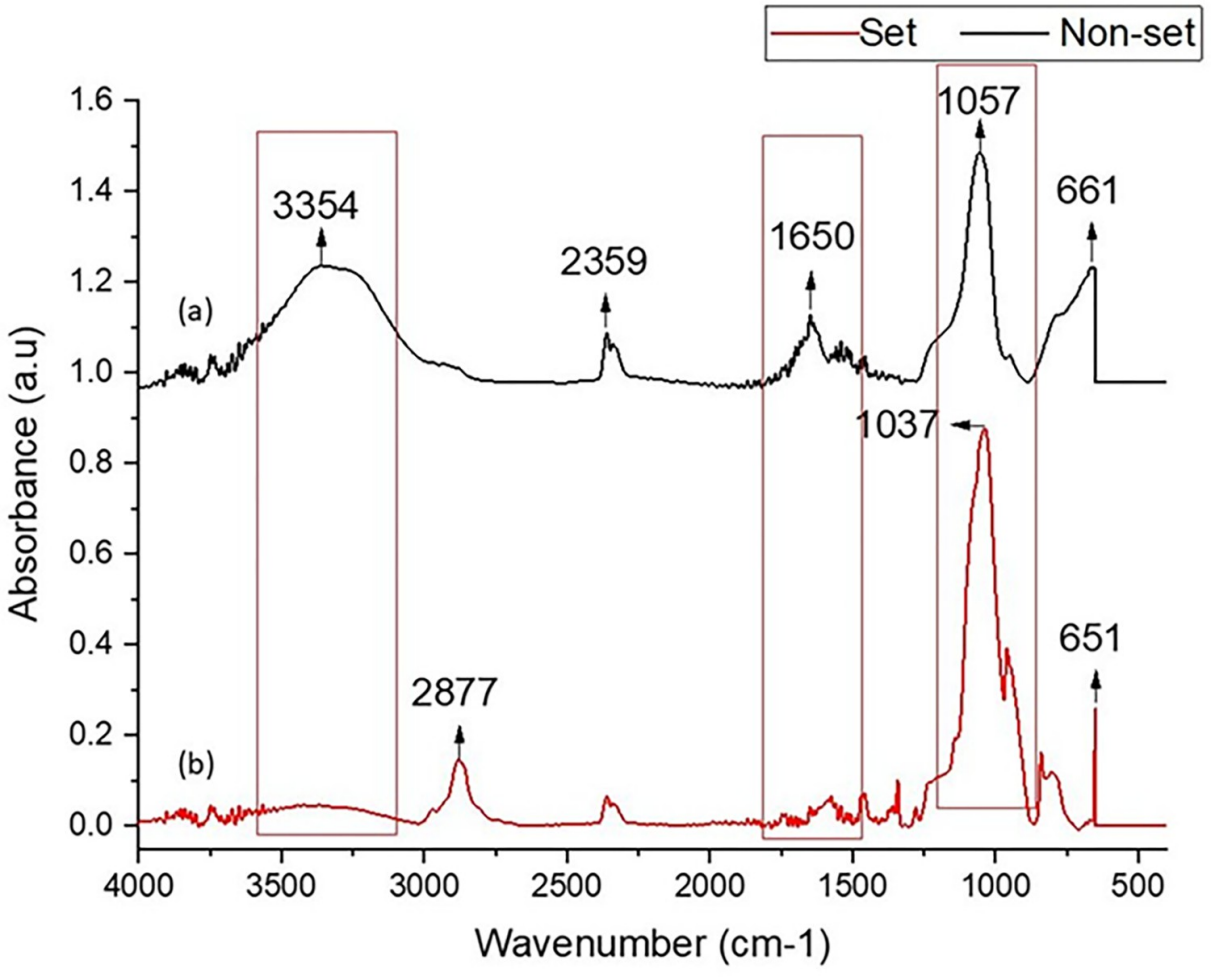
FTIR spectra of (a) non-set and (b) set injectable F-BG showing a reduction in peak height intensities of O-H (stretching peak at 3354 cm^-1^, bending peak at 1650 cm^-1^, P-O and Si-O-Si peaks (1200–900 cm^-1^) after setting.

The comparative FTIR spectra of injectable F-BG before and after setting is shown in Fig 1. A broad band around 3650–3000 cm^-1^ (stretching) and the peak at 1636 cm^-1^ (bending) in spectra of hydrogel (before-setting) showed the presence of molecular water in the form of hydroxyl group (OH^-^). The characteristic peaks of all groups showed a marked increase in peak height after setting except the hydroxyl functional group, which showed a decrease in peak intensity. The stretching symmetric and asymmetric peaks of C-H were seen at 2879 and 2936 cm^-1^, respectively, in the set material. The stretching asymmetric carbonate peak appeared at 1456 cm^-1^ and a reduction in peak intensity was observed after setting at 21°C. The large stretching absorption peak specific to Si-O-Si was seen between 1023 and 1144 cm^-1^ in the set spectra, which was clearly observed as intensified after the setting and overlapping the P-O and was replaced by a shoulder peak at 958 cm^-1^. The band around 1030 cm^-1^ was the triply degenerated peak of phosphate stretching vibration. HPMC showed the presence of a strong characteristic vibration band at 1100 cm^-1^ associated with (C-O), which was overlapped by the sharp stretching peak of the phosphate. The intensity of these peaks was increased due to the molecular rearrangement and due to the reaction and overlapping of other constituents of the injectable material. The peak at 841 cm^-1^ was attributed to the bending asymmetric peak of carbonate. The change in peak intensities before and after the setting of injectable F-BAG have been tabulated in Table 2.

**Table 2:** Peak height differences of set and unset injectable F-BG.

Groups	Peaks (cm^-1^)	Absorbance before setting	Absorbance after setting
O-H (stretching)	3650–3000	0.32	0.03
O-H (bending)	1636	0.166	0.043
Si-O-Si	1023	0.24	0.836
P-O	958	0.163	0.470
CO_3_	872	0.214	0.219

### Morphological analysis

**Fig 2:**
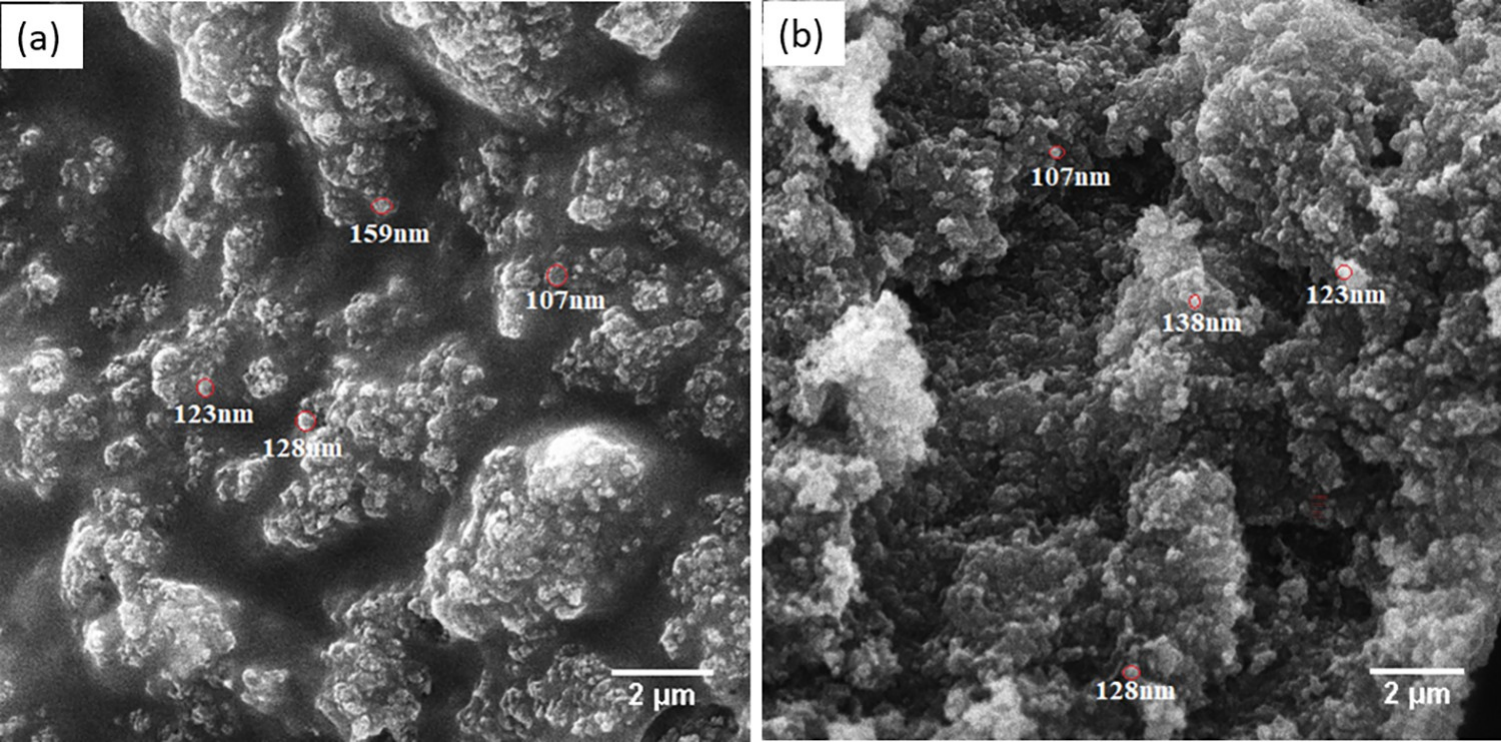
SEM images of the injectable F-BG set at 21°C without ultrasonic scalar (a), and with ultrasonic scalar (b) showing the particles encapsulated in the polymeric network.

SEM images of the set (with and without ultrasonic scalar) injectable material at high magnifications of 10 kx (Fig 2A and 2B) showed the presence of nanoparticles of F-BG in a range of 50–150 nm, which were encapsulated in the polymeric network and formed agglomerates. The glass particles within the polymeric network material were randomly distributed. It was revealed that the densification of the structure occurred due to compaction and that the pore size between the encapsulated particles decreased to a minimum of 220–370 μm (S1 Fig).

### Phase analysis

**Fig 3:**
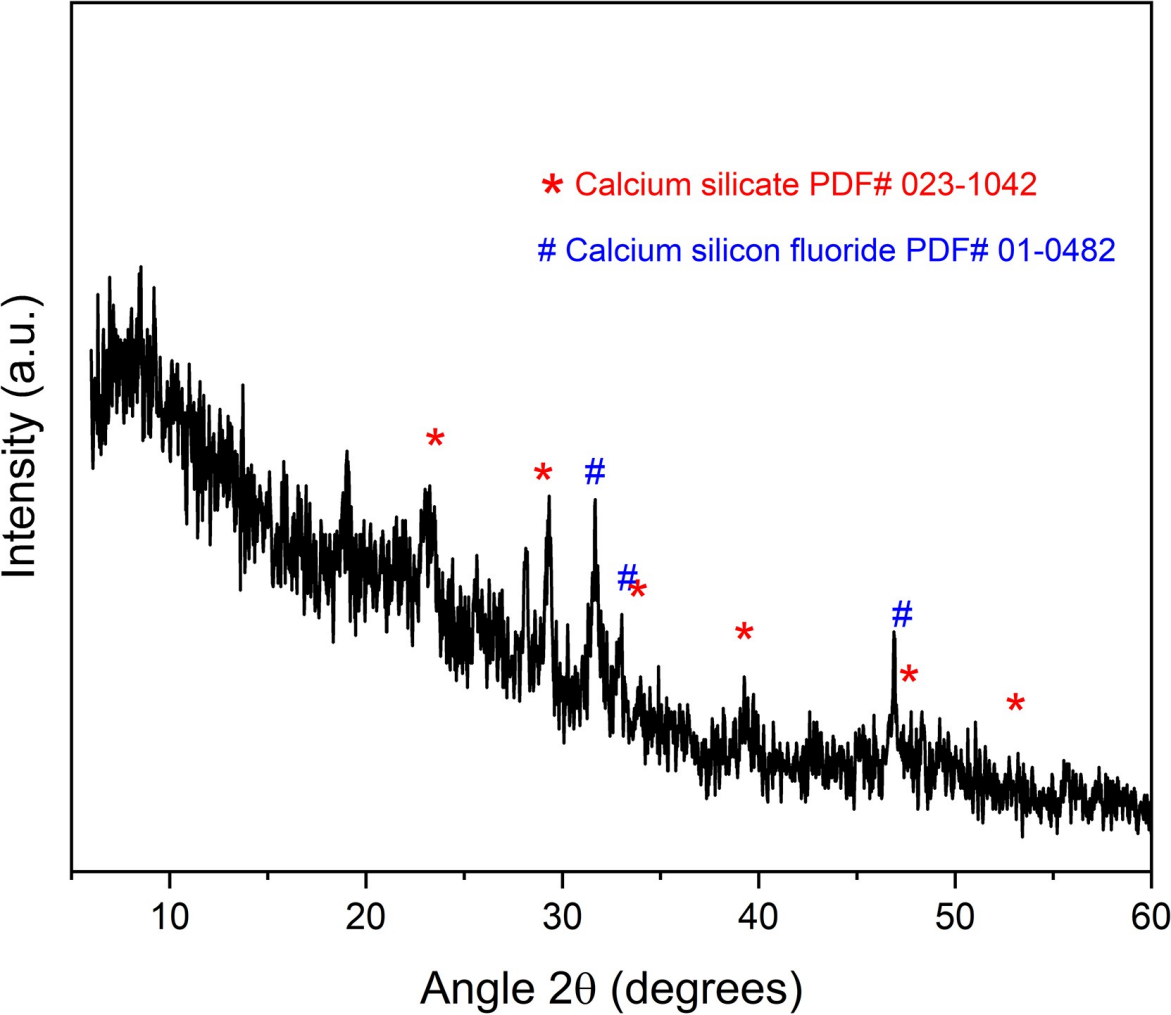
XRD pattern of the set sample of the synthesized injectable F-BG showing the calcium-silicon-fluoride and calcium-silicate phases.

The phase purity and structure of set sample at room temperature 21°C was investigated through XRD analysis and the diffraction pattern is shown in Fig 3. The diffraction pattern showed characteristic peaks of calcium-silicon-fluoride phase (PDF # 001–0482) and calcium silicate phase (PDF # 023–1042) in injectable F-BG.

### Flowability and injectability

Flow analysis indicated that the injectable F-BG demonstrated a flow value greater than 20 mm, which is in agreement with ISO 6876:2012 standards. The mean ± SD flow value of BG-F sealer was 25 ± 3.94 mm.

The mean values of injectability test were found to be 70.3% ± 1.0, 64.3% ± 4.04, and 57% ± 2.0 for 22, 21-, and 23-gauge needles, respectively, as shown in Table 3. The percentage injectability coefficient remained around ≥ 70% in gauge 21 and gauge 22 needle. However, it reduced to 57% in gauge 23 needle with a statistically significant difference (p = 0.003) between the injectability coefficients in all three groups.

**Table 3:** Injectability coefficient of injectable fluoride doped bioactive glass through various gauge needles.

Needle gauge	Needle length (mm)	Internal diameter (mm)	Initial mass (m_o_) mL	Injectability Mean ± SD	P-value
21	32 mm	0.55	1 mL	64.33 ± 4.04.	P = 0.003
22		0.50		70.3 ± 1.52	
23	19 mm	0.40		57 ± 2	

### Bio-adhesion analysis

**Fig 4:**
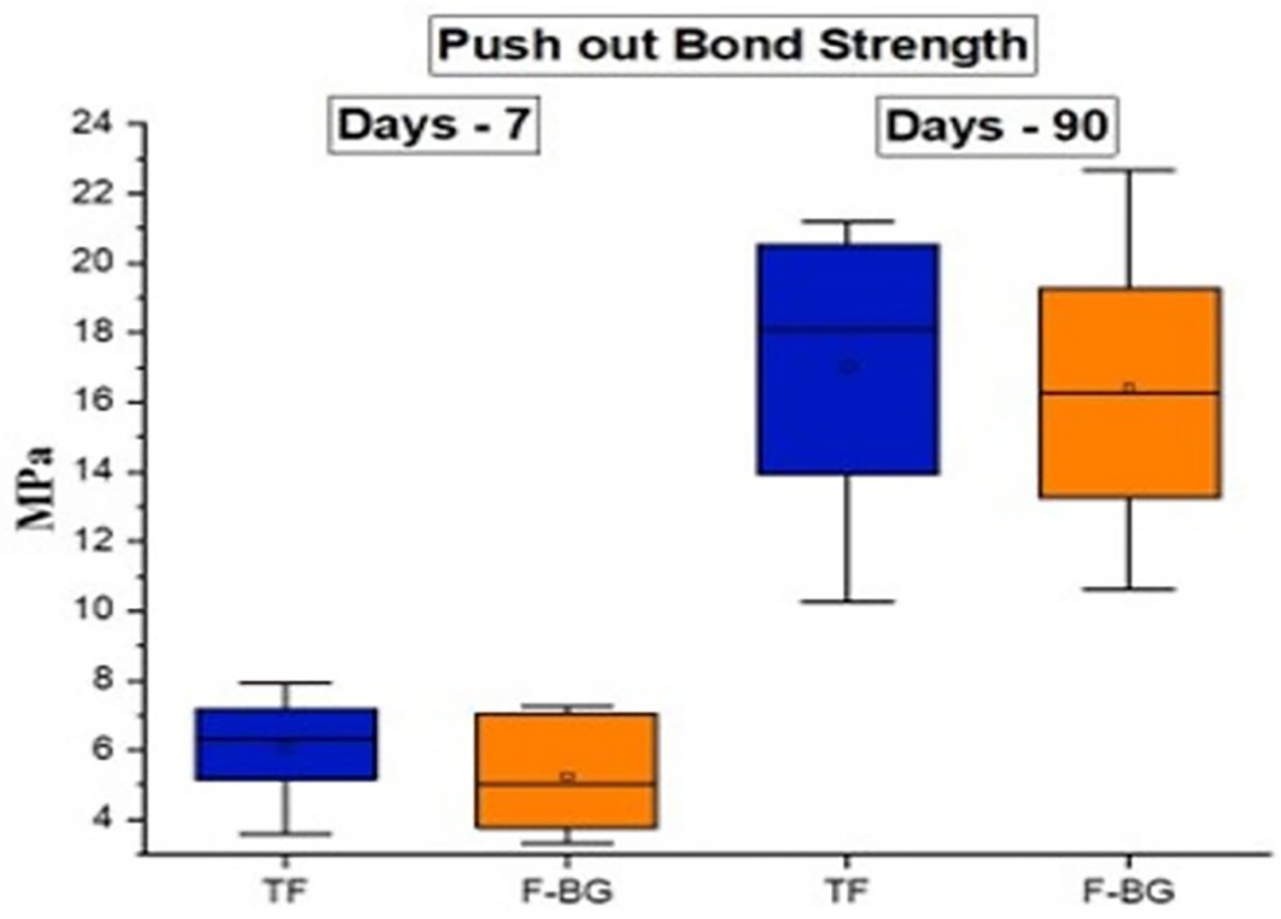
The comparative push-out bond strength analysis of injectable F-BG and TF at days 7 and 90 and non-significant difference was found between the group at each time interval.

**Fig 5:**
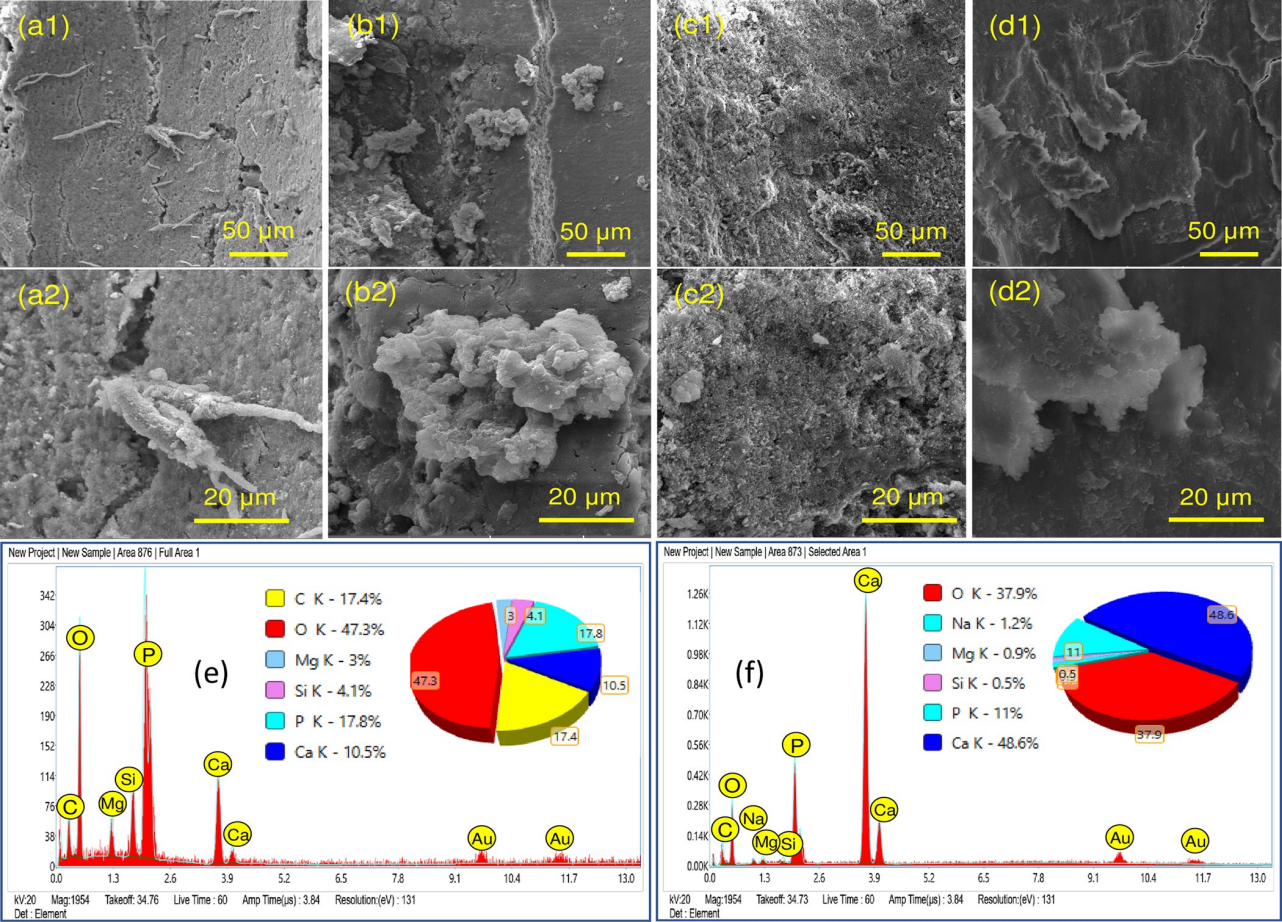
SEM images of injectable F-BG and TF at 500 x (a1-d1) and 2000 x (a2-d2) magnifications showing the presence of particles on the root dentin surface, (a1, a2) SEM images of the injectable F-BG at day 7, (b1, b2) at day 90 (c1, c2) SEM images of TF sealer at day 7and (d1, d2) at day 90. The EDX images of (e) F-BG and (f) TF showing mainly the presence of Ca and P on new apatite layer.

The push-out bond strength (Fig 4) at day 7 showed that TF (6.09 ± 1.53 MPa) had more strength compared to F-BG (5.24 ± 1.63 MPa), however the difference was non-significant (p > 0.05). A similar trend was observed at day 90, where the difference was non-significant between TF (17.01 ± 4.21 MPa) and F-BG (16.38 ± 4.27 MPa). A significant difference (p ≤ 0.05) was observed between days 7 and 90 with both groups. The SEM images (Fig 5) showed the presence of particles on the root dentin surface on day 7. On day 90, both groups showed a new layer on the surface of root dentin. The EDS confirmed the presence of calcium, phosphate, and silicon on the root surface.

### Ion release analysis

**Fig 6:**
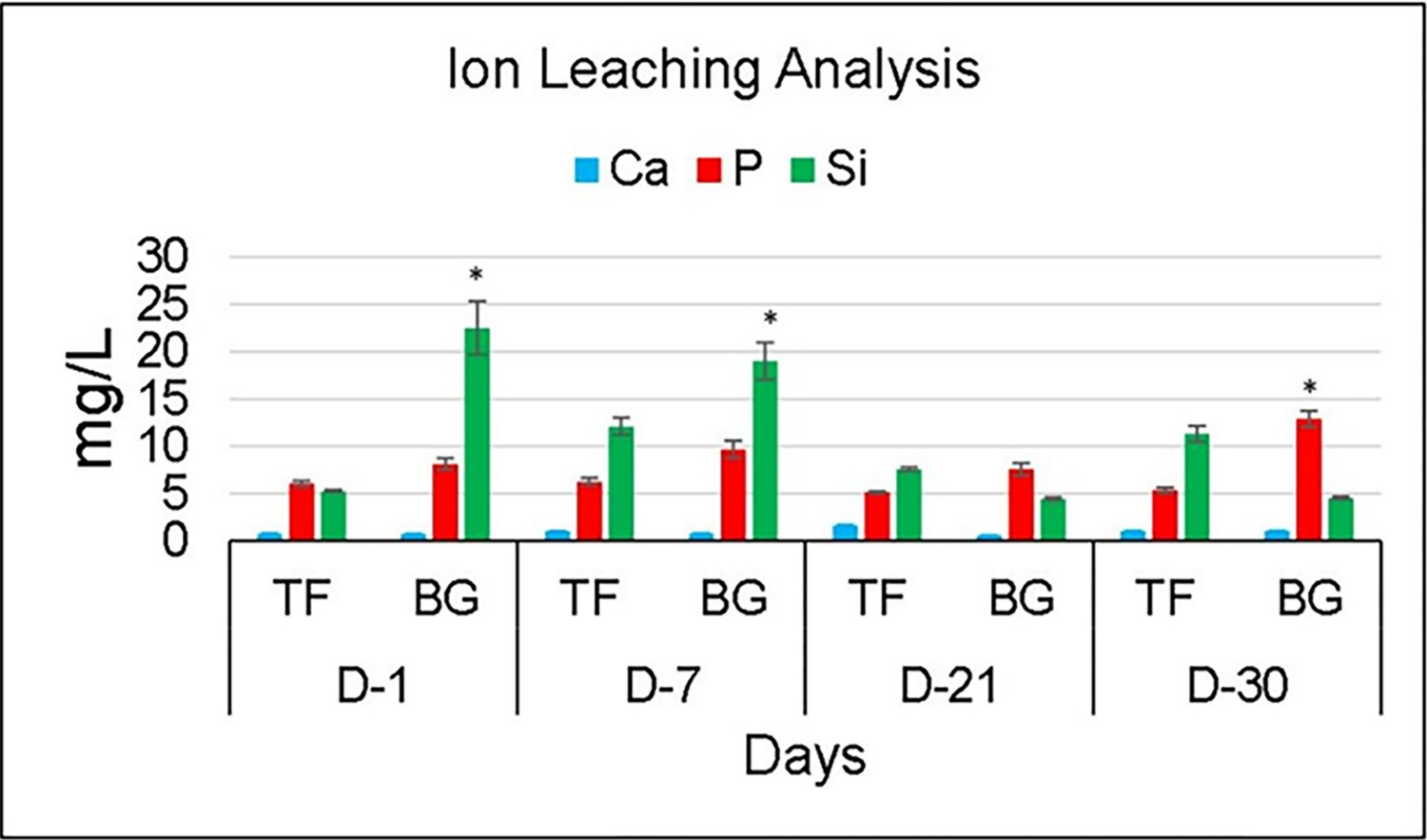
Ion release pattern of F-BG and TF at days 1, 7, 21, and 30. On day 1 and 7, the F-BG showing a statistically higher release of Si than Ca and P. On day 30, there was a statistically higher release of P from F-BG than TF.

A non-linear behavior was observed in ion release analysis (Fig 6), where at day 1, F-BG-based samples showed significantly (p ≤ 0.05) high release of Si ions (22.5 ± 2.82 mg/L) compared to P (8.12 ± 0.59 mg/L) and Ca ions (0.767 ±0 .009 mg/L). The P ion release was significantly (p = 0.036) high compared to Ca. However, TF-based group showed non-significant difference (p = 0.113) between P (6.06 ± 0.28 mg/L) and Si (5.27 ± 0.13 mg/L) ions, whereas significant difference (p = 0.023) was observed between P and Ca ion (0.77 ± 0.009 mg/L). On day 7, F-BG behavior was similar to day 1, however, TF showed significantly more release of Si ion compared to P (p = 0.037) and Ca (p = 0.038). It was found that with time, the release pattern of Si in F-BG group was reduced. However, TF showed non-linear behavior on days 21 and 30. On day 30, for F-BG release of Si ion was significantly (p = 0.012) lower than day 1, however, more release of P (p = 0.022 and Ca (p = 0.005) ions was observed.

#### Fluoride release analysis

**Fig 7:**
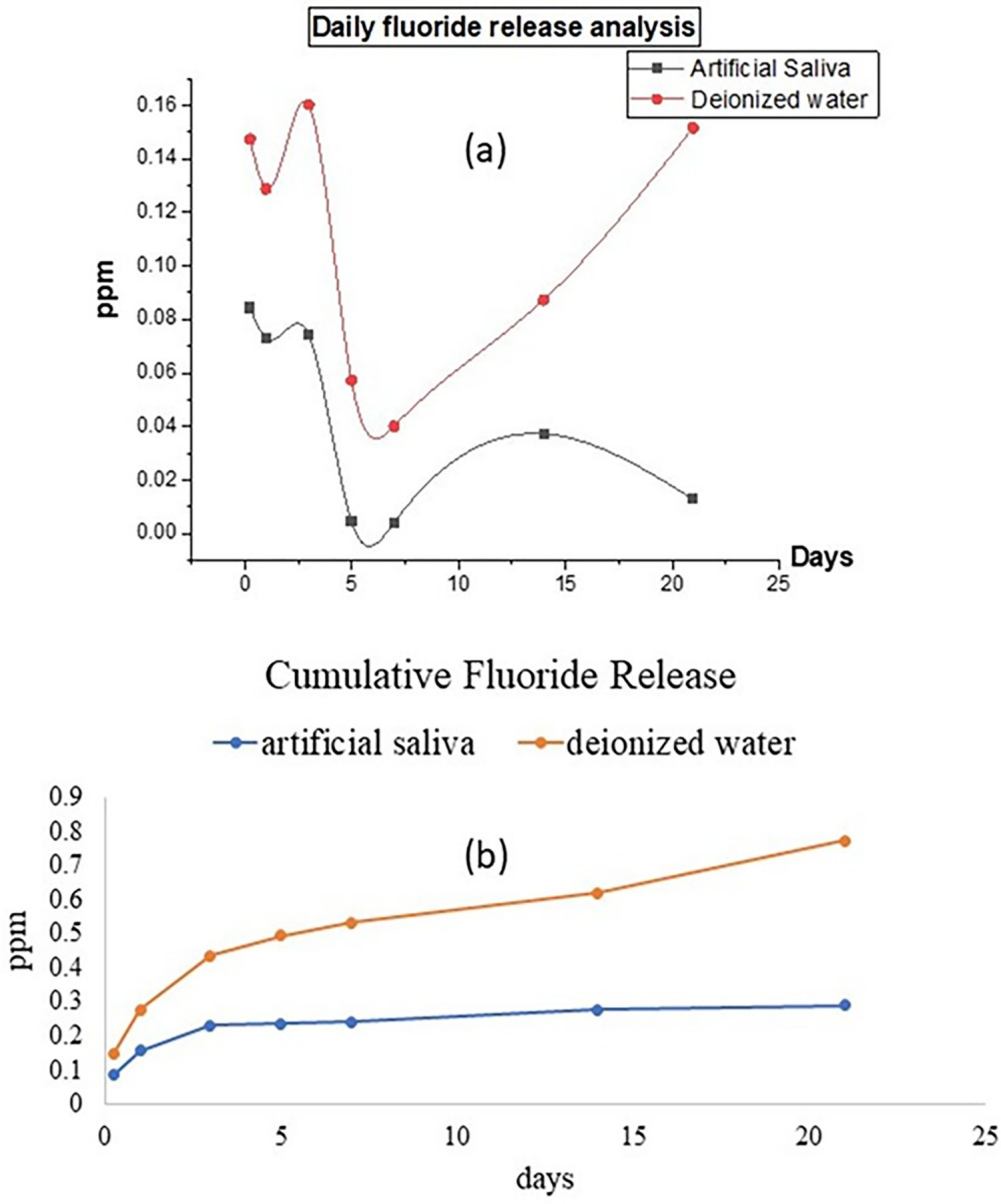
(a) Daily fluoride release and (b) cumulative fluoride release in artificial saliva and deionized water at days 1, 3, 5, 7, 14, and 21.

Fluoride release analysis of the injectable F-BG sample showed that the ion release was greater in deionized water as compared to artificial saliva at all-time intervals. The mean daily values of the injectable F-BG in both deionized water and artificial saliva are plotted against time in Fig 7A. The six hourly fluoride ion release in deionized water was 0.147 ± 0.054 ppm. On day 1, the release was still high (0.129 ± 0.020 ppm), reaching a maximum level of 0.160 ± 0.037 ppm at day 3. After the third day, the ion release reduced to a value of 0.057 ± 0.023 ppm on day 5 and further decreased with a mean value of 0.040 ± 0.026 ppm on day 7. On day 14, the release was found to be increased (0.087 ± 0.049 ppm) and reaching further elevated levels of 0.151 ± 0.039 ppm by day 21. There was a statistically significant difference (p ≤ 0.05) between the two groups at all intervals. Fluoride release in deionized water was found to be significantly higher (p ≤ 0.05) than in artificial saliva at all time intervals, the details are mentioned in Table 4.

**Table 4:** The mean daily fluoride release (ppm) of injectable F-BG in deionized water and artificial saliva with standard deviation and p- value.

Time interval	Deionized water (ppm)	Artificial saliva (ppm)	P- value
**6 h**	0.147 ± 0.054	0.084 ± 0.043	0.034
**Day 1**	0.129 ± 0.020	0.073 ± 0.014	0.000
**Day 3**	0.160 ± 0.037	0.074 ± 0.032	0.001
**Day 5**	0.057 ± 0.023	0.005 ± 0.001	0.001
**Day 7**	0.040 ± 0.026	0.004 ± 0.001	0.011
**Day 14**	0.087 ± 0.049	0.037 ± 0.024	0.040
**Day 21**	0.151 ± 0.039	0.013 ± 0.005	0.000

In artificial saliva, at 6 h, the ion release was 0.084 ± 0.043 ppm. On day 1, ion release was 0.073 ± 0.014 ppm, which remained almost constant on day 3 (0.074 ± 0.032 ppm). Subsequently, the release exhibited a decreasing trend, reaching mean release of 0.005 ± 0.001 ppm and 0.004 ± 0.001 ppm on days 5 and 7, respectively. A pattern similar to deionized water was found for artificial saliva after day 7 as the release at day 14 became higher with a mean value of 0.037 ± 0.024 ppm, whereas it finally decreased at day 21 (0.013 ± 0.005 ppm). A cumulative fluoride ion release of 0.77 ppm was observed for deionized water, and that of 0.29 ppm was observed for artificial saliva at the end of 21 days and is shown in Fig 7B.

## Discussion

It is desirable that the root canal sealer should be capable of creating a direct bond with the dentin. Bioceramics-based materials have gained interest among endodontists, not only as a repairing material [38] but also as a sealer [39]. Forming an apatite layer can help create a monoblock system with sealer and tooth surface. Subsequently, it can improve the bond strength of the material. Conventional bioceramics-based sealers showed clinical disadvantages such as difficulty in handling, a high pH during setting, long setting times, and that hardening requires sufficient moisture [40,41]. To overcome these drawbacks, an experimental injectable fluoridated-bioactive glass-based canal sealer was developed in this study. The injectable fluoridated-bioactive glass was synthesized using the Pluronic F127 and HPMC as a carrier. The fluoride ions are assumed to have fused into the glass network in the form of calcium fluoride during the synthesis of bioactive glass powder and were released in a periodic manner when the samples were immersed in specific media.

The spectroscopic analysis confirmed the synthesis of cross-linked injectable F-BG showing chemical groups from both the bioactive glass powder and the polymer. The FTIR spectra of F-BG showed the changes in intensities of the peaks before and after setting. Before setting OH^-^ from the water appeared as a broad band between 3650 to 3000 cm^-1^. This intensity of the broad band was reduced as the material set, eliminating the water content from the injectable F-BG. In comparison, the intensities of the spectral band of Si-O-Si and P-O were increased after the setting of the material, which might be due to the molecular rearrangement, reaction, and overlapping of other constituents of the injectable material. It is expected that a change in the structure of the injectable F-BG was likely to occur due to some non-covalent bonding between the terminal OH groups of the polymer and the Si of the F-BG.

SEM images of BG showed that the bioactive glass particles were spherical and randomly distributed, coalesced together to form agglomerates in the polymeric network. The images revealed a porous structure, and the agglomeration of the BG particles probably occurred due to the large surface area of the nanoparticles in the nanometer range. This coalescence of the particles might be due to the Vander Waal forces that act as a major driving force [42]. According to the SEM images, the injectable F-BG showed a porous structure, and it would favor the possibility of new apatite formation with the intended use of this injectable bioactive material in endodontics. The x-ray diffractogram confirmed the amorphous nature mainly with few low intense peaks indicated some crystalline phases that might have formed during the sintering procedure and also showing nanocrystalline polymer. The main phases observed in the XRD pattern were nanocrystals of calcium silicate and calcium silicon fluoride. The samples showed the presence of a fluorite phase in the form of calcium silicon fluoride, which would crystallize to fluorapatite rather than the wollastonite phase. A less crystalline phase is more desirable for an endodontic application due to the increased ions’ dissolution rate, subsequently forming the apatite layer.

It is important to create standardized procedures to evaluate the properties of the new and gold standard of endodontic materials such as root canal sealers. No specific standard of root canal sealer setting time is reported. However, the operator needs an adequate working time to place the material in the root canal. In the present study, the setting time of the injectable F-BG was in an acceptable range (more than 30 min). The setting time of the injectable bioactive glass was measured at 21°C with and without ultrasonic scalar and at 37°C. In this study, the lowest mean value of setting time was observed for the ultrasonic scalars-based group. The effects of application of ultrasonic waves to the surface of the cement induce a quick removal of water due to energy of ultrasonic waves [43,44]. The ultrasonic excitation promotes a more homogenous mixture between the glass particles, increasing the particle rate dissolution, ionic diffusion through the liquid, and accelerating the crosslinking [45]. Ultrasound waves to the material were applied for 20 s and a rest period of 5 s was given in between the intervals to avoid heating of the material. The amount of heat generated from the scalar and its effect on the setting characteristics of the material is beyond the scope of this study, however it is assumed that the heat would accelerate the setting of the material. The effects of heat on the polymeric constituents also need to be evaluated in future study as the glass transition temperature of the polymer is very low (around 50°C).

Flowability is an important consideration for the usage of sealers in the root canal. The greater penetration capability of sealers through dentinal tubules lead to greater ability to trap microorganisms and inhibit their growth [46,47]. A high flow rate is considered an advantage to the sealer; however, it should be limited to avoid extrusion outside the canal in the preapical tissues [46]. The flowability of experimental injectable F-BG is in accordance with previously reported flowability of the commercially available injectable sealers [48,49]. The obtained value of this study is in accordance with ISO 6876:2012 standards. Along with the flowability, injectability is an important characteristic of the endodontic sealers, which allow them to fill root canal systems in all their complexity. The injectability of F-BG material was measured by the percentage of the extruded paste from the syringe fitted with the needle. The results showed that the F-BG performed well through all needle sizes. A slight decrease in flow was observed in the 23 G needle. Injectability is the major performance parameter of any parenteral dosage form and can be affected by the needle geometry, i.e., length, inner diameter, shape of the opening, as well as the surface finish of the syringe [50]. The ease in injectability measurement is important in clinical application if the material is injected by hand and the injection force should be limited to 200 N. There is no common understanding regarding the bio-cements about the meaning of injectability, and many authors relate injectability to the injection force that has to be applied on a syringe to inject the cement paste [51]. As in this study, three different gauges of needles were used i.e., gauge 21 (internal diameter; 0.55 mm, length; 1 x 1/4ʺ), gauge 22 (internal diameter; 0.50 mm, length; 1 x 1/4ʺ) and gauge 23 (internal diameter; 0.40 mm, length; 3/4ʺ). The results showed that the decrease in internal diameter of the needle from 0.55 to 0.40 with respect to needle gauges affected the injectability of the pastes.

The adequate adhesion of the sealers can enhance the sealing ability and inhibit apical and coronal leakage. Many factors can influence adhesion, such as the chemical structure, interaction with root dentin, smear layer presence, and the non-obliterated dentinal tubules [35]. The bond strength of experimental F-BG was in comparison with the commercially available TF injectable sealer. A non-significant difference was observed between both groups. A significant difference was observed between day 7 and 90. The high bond strength after 90 days could be due to the enhanced linkage of sealers with root dentin. It was confirmed with the SEM analysis that showed the presence of a new apatite layer and depositing hydroxyapatite that chemically bonds to the tooth structure. There was a presence of attached nanoparticles initially, and after 90 days, a newly formed apatite layer was observed. Several studies have been reported in literature over the years that have proven the mineralization potential of dentin with the use of bioactive glass [52,53]. There is a compositional difference between the commercial and the experimental injectable sealers. The TF is based on calcium silicate, calcium phosphate monobasic, zirconia, and tantalum oxide, whereas the experimental injectable is based on fluoridated bioactive glass. The authors could not find any commercial bioactive glass-based endodontic sealer, therefore, TF was used as a control group. The Ca and Si ions are the main components of bioactive glass- and calcium silicate-based materials. The ICP-OES data also confirmed the release of ions, and it was found that the F-BG showed abrupt release of Si on day 1, whereby the release pattern was increased up to day 7. The maximum release of Si was observed compared to Ca and P. The high release could be due to higher molar % of Si in the composition of injectable bioactive glass. The mechanism of bioactivity is directly associated with the release of Si, Ca, and P and is well-reported in the literature [54]. The hydrolysis of Si-O-Si might have occurred due to the interaction with water [55,56]. To confirm the release pattern of fluoride, the ion release from the experimental injectable F-BG was studied in two media i.e., deionized water and artificial saliva. As TF injectable does not contain fluoride ions in its composition, therefore, it was not included in the study. Artificial saliva was used to closely mimic the natural environment of the oral cavity. The release of fluoride ions was found to be greater in deionized water (cumulative release 0.771 ppm) as compared to artificial saliva (cumulative release 0.290 ppm). This can be explained in terms of the compositions of the two media. Artificial saliva consists of various free ions, calcium among which is the most significant. The presence of calcium ions in the media can modify the release of fluoride ions due to the formation of calcium fluoride. This calcium fluoride precipitates over the surface hampering the release of fluoride ions [57]. Apart from calcium, the presence of phosphates also interferes with the fluoride ion release [58]. These results are in accordance with the previous studies [36,58,59] where the researchers used different restorative dental materials i.e., glass ionomer cements, resin-modified glass ionomer cements, and giomers to analyze fluoride release in these two media. The release of ions is also affected by the pH of the solutions. Fluoride ion release has been reported to be high in an environment of low pH value. The pattern of ion release in injectable bioactive glass also showed an initial burst of ions in the first three days due to the extra framework fluoride present in the bioactive glass, and then there is a drop in release after day 3, which is because of the aging and disintegration of material with time. It can be assumed that a layer of fluorapatite may have formed on the surface of the glass. This apatite layer must have reduced the solubility of the glass and interfered with the ion release from the surface. The phosphate and fluoride ions released into the solution would have been used in the formation of this fluorapatite. After a certain interval of time, the particles disperse and scatter in the media and cause an increase in the surface area of the glass, as seen after day 14. Another possible reason for this could be the recharging phenomenon, as in the case of GICs. The glass particles might have taken up the free fluoride ions present in the oral environment and would have caused the increase of fluoride ions after day 14. The concentration of fluoride on day 1 (0.14 ppm) increased to 0.16 ppm and 0.15 ppm on day 3 and 5, respectively, in deionized water, portraying that the release of fluoride ions from this injectable F-BG is a continuous phenomenon and does not follow a decreasing gradient. This property makes this injectable material a very interesting material to be used in dental applications such as an injectable root canal sealant with a continuous fluoride releasing ability would be a very attractive material to be used in root canal treatments.

## Conclusion

Within the limitation of this study, it is concluded that FTIR analysis revealed the characteristic peaks of bioactive glass in an injectable form that confirmed the successful incorporation of these particles within the polymeric network. In SEM analysis, the bioactive glass particle was spherical and distributed in the polymeric network, coalesced together to form agglomerates, and revealed a porous structure. XRD showed the amorphous phase and the presence of two phases: calcium silicon fluoride and calcium silicate. The clinical acceptable setting time was achieved, where an ultrasonically assisted setting showed the shortest setting time. The injectability coefficient remained around ≥ 70% in needle gauge sizes 21 and 22 and was reduced to 57% in size 23. Adequate bond strength was achieved, the values were increased with time, and continuous release of Ca, P, and Si ions was observed. The cumulative fluoride release was observed from the injectable F-BG. Despite numerous scientific literatures on injectable bioactive glass, much research is needed to better understand this material’s physical, chemical, and biological properties.

## Supporting information

S1 Fig SEM images of the injectable F-BG set at 21°C with ultrasonic scalar. (TIF)

S1 File (PDF)
