# Supplementary figures and images for: Synthesis and analyses of injectable fluoridated-bioactive glass hydrogel for dental root canal sealing
Source: PLoS One. 2023 Nov 27;18(11):e0294446. doi: 10.1371/journal.pone.0294446 (PMC10681180; doi:10.1371/journal.pone.0294446)

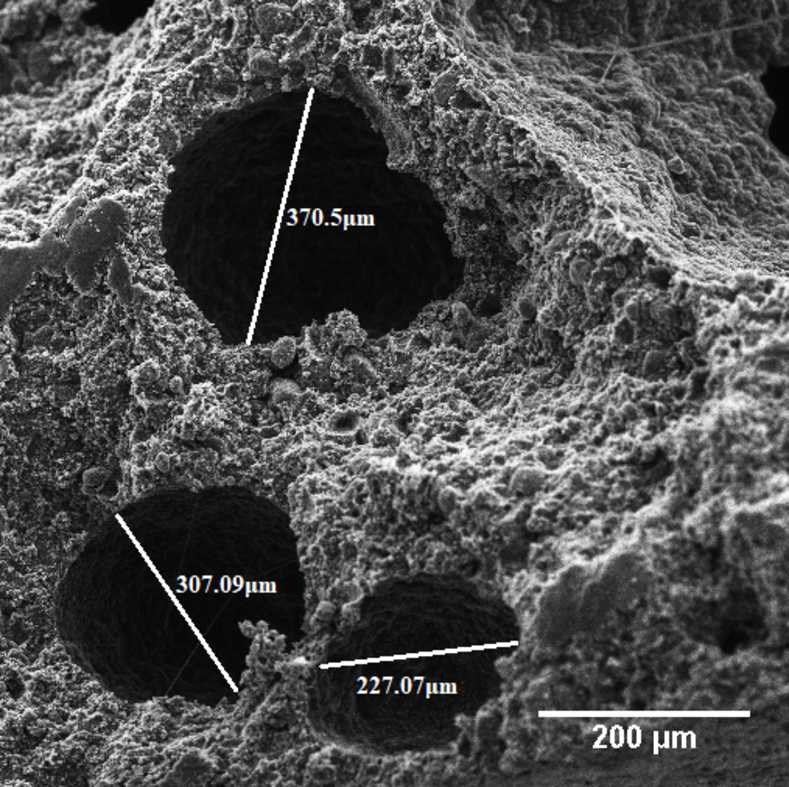

Supplement: S1 Fig — (TIF) [file pone.0294446.s001.tif]
