# Supplementary material for: Synthesis and analyses of injectable fluoridated-bioactive glass hydrogel for dental root canal sealing
Source: PLoS One. 2023 Nov 27;18(11):e0294446. doi: 10.1371/journal.pone.0294446 (PMC10681180; doi:10.1371/journal.pone.0294446)
Supplement: S1 File — (PDF) [file pone.0294446.s002.pdf]

Supporting information

for

Synthesis and analyses of injectable fluoridated-bioactive glass hydrogel for dental root canal sealing

Figure S1: SEM images of the injectable F-BG set at 21°C with ultrasonic scalar
